# The Improvement of Fluorescence In Situ Hybridization Technique Based on Explorations of Symbionts in Cicadas

**DOI:** 10.3390/ijms242115838

**Published:** 2023-10-31

**Authors:** Zhi Huang, Dandan Wang, Jinrui Zhou, Hong He, Cong Wei

**Affiliations:** 1 Key Laboratory of Plant Protection Resources and Pest Management of the Ministry of Education, College of Plant Protection, Northwest A&F University, Yangling 712100, China; huangzhi@nwafu.edu.cn (Z.H.); wangdandan@ioz.ac.cn (D.W.); jinruizhou@nwafu.edu.cn (J.Z.); 2 College of Forestry, Northwest A&F University, Yangling 712100, China

**Keywords:** heat shock, helpers, oligonucleotide probes, inaccessibility

## Abstract

Fluorescence in situ hybridization (FISH) with rRNA-targeted oligonucleotide probes is widely used for the identification of microbes in complex samples, but it suffers from some limitations resulting in the weak or even absence of fluorescence signals of microbe(s), which may lead to the underestimation or misunderstanding of a microbial community. Herein, we explored symbionts in the bacteriomes and fat bodies of cicadas using modified FISH, aiming to improve this technique. We initially revealed that the probes of *Candidatus* Sulcia muelleri (*Sulcia*) and the yeast-like fungal symbiont (YLS) are suitable for detection of these symbionts in all cicadas and some other species of Auchenorrhyncha, whereas the probe of *Candidatus* Hodgkinia cicadicola (*Hodgkinia*) is only suitable for detection of *Hodgkinia* in a few cicada species. The fluorescence signal of *Sulcia*, *Hodgkinia* and YLS exhibited weak intensity without the addition of unlabeled oligonucleotides (helpers) and heat shock in some cicadas; however, it can be significantly improved by the addition of both helpers and heat shock. Results of this study suggest that heat shock denaturing rRNA and proteins of related microbe(s) together with helpers binding to the adjacent region of the probe’s target sites prevent the re-establishment of the native secondary structure of rRNA; therefore, suitable probe(s) can more easily access to the probe’s target sites of rRNA. Our results provide new information for the significant improvement of hybridization signal intensities of microbes in the FISH experiment, making it possible to achieve a more precise understanding of the microbial distribution, community and density in complex samples.

## 1. Introduction

Traditional methods of microorganism identification were mainly based on morphological, biochemical and metabolic characteristics of the individual microbe(s), which could be successfully isolated using culture-based methods [1]. One of the major limitations of culture-based methods is that they are quite time-consuming for microbe identification and, therefore, it is not an ideal method for the rapid identification of microbes in complex environmental samples [2,3]. Additionally, cultured microorganisms only account for <1% of the total environmental microbes, and the majority of microbes (e.g., most animal symbionts) are currently uncultivable under laboratory conditions [1,3,4,5,6]. However, the accurate identification of these uncultivable microbes still remains a challenging task according to their morphological characteristics under histological and/or ultrastructural microscopy. Over the past three decades, fluorescence in situ hybridization (FISH) with rRNA-targeted oligonucleotide probes has become a cultivation-independent method for the in situ analysis of the composition and dynamics of microbial communities in complex samples, such as animal tissues and clinical specimens [2].

The small subunit ribosomal RNA (rRNA) is the most commonly used target molecule for FISH experiments, which include the 16S rRNA of prokaryotes and 18S rRNA of eukaryotes [1,2,3]. One of the beneficial properties of using rRNA molecules for FISH is their genetic stability and the high copy number of them. Microbial cells require a lot of ribosomes for translation, and thus, these rRNA molecules of ribosomes are usually amplified to numbers ranging from a few hundred to 100,000 in each cell [2,7]. Additionally, the evolutionary conservation of rRNA genes is patchy but is generally much higher than that of most protein-encoding genes [2]. Therefore, rRNA genes are more suitable than other genes for the rapid and accurate identification of microbe(s) by FISH with rRNA-targeted oligonucleotide probes [1,2,3]. A systematic study of the secondary structure of rRNA revealed that a stem refers to consecutive base pairs with no unpaired bases separating them, and a hairpin loop refers to consecutive unpaired bases that are closed by one base pair [8]. The fluorescent probe is 15–25 nucleotides in length, and it is usually labeled at the 5′ end with a fluorescence dye such as Cy3 and Cy5 [1,2,3]. Oligonucleotide probes can be designed based on the rRNA gene sequences of target species using several primer design software (e.g., Primer 5.0) and web-based services according to the guidelines [1,9,10,11]. It is also feasible to search for the rRNA-targeted oligonucleotide probes in publically available probe databases (e.g., probeBase) or published articles [1,9,10,11].

To date, the FISH technique has become an extremely valuable tool for the detection of microbes in complex environmental samples, particularly for invertebrate animal tissues. The FISH technique has been widely used to explore the distribution and transmission process of symbionts in insects, especially for sap-feeding insects in the suborder Auchenorrhyncha of Hemiptera [12,13,14]. The Auchenorrhyncha, which includes spittlebugs, treehoppers, leafhoppers, planthoppers and cicadas, has been referred to as the ‘fairyland of symbiosis’ [15,16]. It is generally thought that the auchenorrhynchan ancestor developed an intimate symbiosis with a *Bacteroidetes* currently known as “*Candidatus* Sulcia muelleri” (referred to as *Sulcia*) and a coresident betaproteobacterium, which can supplement their nutritionally unbalanced diet with essential nutrients including essential amino acids, vitamins and cobalamin [14,16,17]. Fluorescence microscopy revealed that some cicadas harbored *Sulcia* and *Candidatus* Hodgkinia cicadicola (referred to as *Hodgkinia*) in the peripheral bacteriocytes and central bacteriocytes of the bacteriomes, respectively [12,18,19,20]. However, in many cicada species, *Hodgkinia* has been replaced by a yeast-like fungal symbiont (YLS) [21,22,23]. Fluorescence and histological microscopy showed that YLS cells are only harbored in the fat bodies among the majority of *Hodgkinia*-free cicada species (e.g., *Cryptotympana atrata*), whereas they are harbored in both the fat bodies and bacteriome sheath in a few *Hodgkinia*-free cicada species, such as *Hyalessa maculaticollis* and *Graptopsaltria tienta* [21,22,23].

However, the FISH technique still suffers from some limitations, including limited cell membrane permeability, low cellular ribosome content and inaccessibility of the probe binding sites, which can result in the weak or even absence of fluorescence signal intensity of targeted microbe(s). Therefore, it may eventually lead to the underestimation or misunderstanding of the density and distribution of related microbe(s) in the complex samples. For example, the distribution of YLS was not determined in leafhoppers *Ledropsis discolor*, *Ledra auditura* and *Tituria angulata* by fluorescently labeled oligonucleotide probe under the fluorescence microscopy, although it can be identified in the fat bodies of the above three leafhoppers under histological microscopy [24]. Fluorescence microscopy revealed that YLS exhibited low hybridization signals in the bacteriome sheath but no hybridization signals in the fat bodies of *Graptopsaltria nigrofuscata*, and concluded that YLS cells were only harbored in the bacteriome sheath of this cicada [21]. Another example is that YLS cells exhibited weak hybridization signals in the bacteriome sheath and fat bodies of *G. tienta*, leading to the underestimation of the YLS density in these two types of tissues [22]. It has been shown that a modified FISH technique through the addition of both unlabeled oligonucleotides (helpers) and heat shock can greatly enhance FISH signal intensities of YLS in cicadas *Karenia caelatata* and *Tanna* sp. [23] Whether YLS is distributed in the fat bodies of *G. nigrofuscata* and its density (if present) remains questionable. The distribution of facultative symbionts *Arsenophonus* and *Rickettsia* in cicadas *Eopycna repanda* and *Platypleura kaempferi* have been clearly clarified based on fluorescence microscopy, whereas previous studies using the FISH technique failed to visualize the fluorescence signals of other facultative symbionts including *Spiroplasma* and *Sodalis* that could be detected by other methods, such as diagnostic PCR amplification. Therefore, a modified FISH technique may contribute to the improvement of the hybridization signal intensity of microbes, which provides more precise information about the distribution patterns, microbe density and transmission process of symbionts in insects.

In this study, we initially explored the similarity between rRNA-targeted oligonucleotide probes and 16S rRNA/18S rRNA gene sequences of targeted microbes in cicadas and other representative auchenorrhynchan insects. Then, we compared the FISH signal intensities of *Sulcia*, *Hodgkinia* and YLS under the control group and three different treatment groups using seven representative cicada species, *K. caelatata*, *C. atrata*, *Macrosemia insignis*, *H. maculaticollis*, *E. repanda*, *Eopycna coelestia* and *P. kaempferi*. We explored whether the addition of heat shock and helpers can contribute to the enhancement of FISH signal intensities of *Sulcia*, *Hodgkinia* and YLS in these cicadas. We further predicted the secondary structure of the 16S rRNA/18S rRNA gene sequence of related symbionts in these cicada species, which may be responsible for the FISH signal intensity of symbiont(s). The results may provide new information for the improvement of hybridization signal intensities of microbes in the FISH experiments.

## 2. Results

### 2.1. Similarity between rRNA-Targeted Oligonucleotide Probes and 16S rRNA/18S rRNA Gene Sequence of Targeted Microbes in Cicadas and Other Auchenorrhynchan Insects

**Figure 1:**
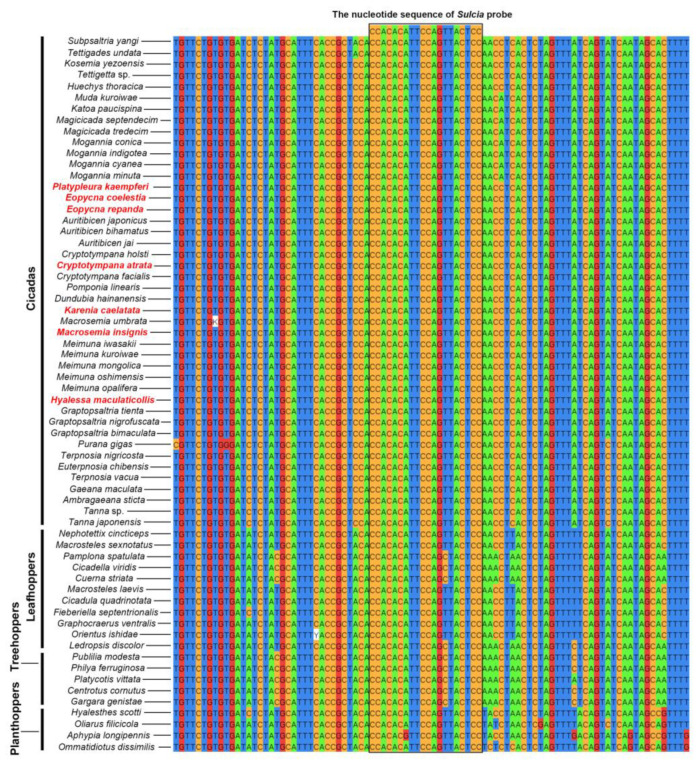
Similarity between the nucleotide sequence of *Sulcia* probe and 16S rRNA gene sequences of *Sulcia* in cicadas and other representative auchenorrhynchan insects. Different color represents distinct DNA bases. Red font represents the cicada species used in this study.

The results of sequence similarity revealed that the nucleotide sequence of *Sulcia* probe used in this study shows 100% similarity with the 16S rRNA gene sequences of *Sulcia* symbionts in all cicadas (Figure 1, Table 1 and Table S1). The nucleotide sequence of *Sulcia* probe exhibits 100% similarity with the 16S rRNA gene sequences of *Sulcia* symbionts in some leafhoppers and planthoppers (e.g., *Macrosteles sexnotatus*), whereas it shows 95% similarity with the 16S rRNA gene sequences of *Sulcia* symbionts in some treehoppers (e.g., *Publilia modesta*), leafhoppers and planthoppers (Figure 1, Table 1 and Table S2). 

**Figure 2:**
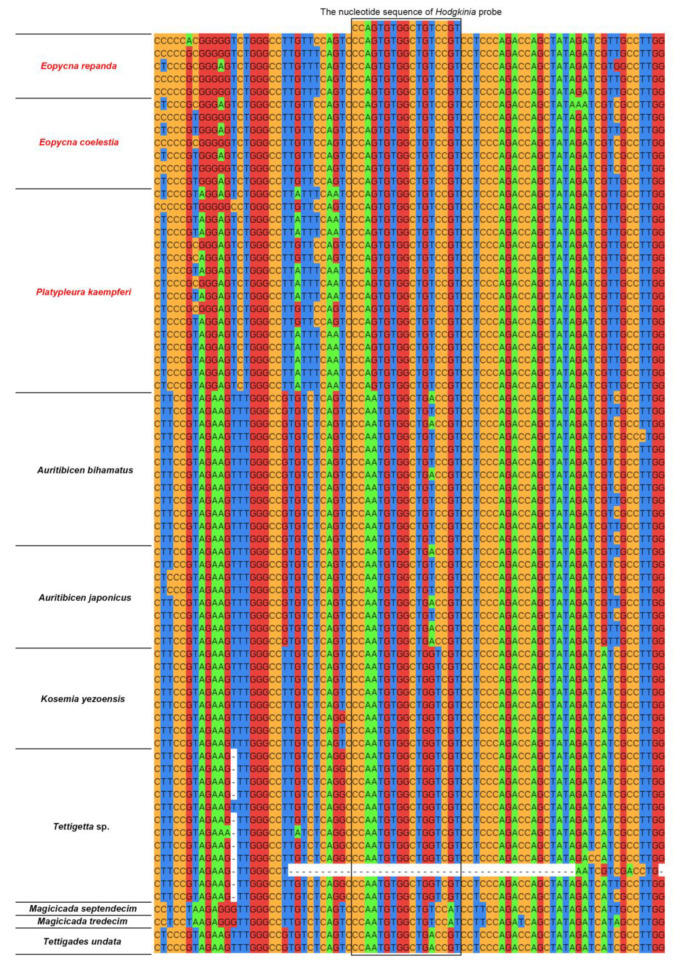
Similarity between the nucleotide sequence of *Hodgkinia*-Ple probe and 16S rRNA gene sequences of *Hodgkinia* in cicadas. Different color represents distinct DNA bases. Red font represents the cicada species used in this study.

The nucleotide sequence of the *Hodgkinia*-Ple probe exhibits 100% similarity with the 16S rRNA gene sequences of *Hodgkinia* symbionts in cicadas of the tribe Platypleurini, including *P. kaempferi*, *E*. *coelestia* and *E*. *repanda* (Figure 2, Table 1 and Table S1). However, the nucleotide sequence of *Hodgkinia*-Ple probe shows an extremely low similarity (82.35% or 88.24%) with the 16S rRNA gene sequences of *Hodgkinia* symbionts in some cicada species, including *Tettigades undata*, *Kosemia yezoensis*, *Tettigetta* sp., *Magicicada septendecim* and *Magicicada tredecim*. Additionally, the nucleotide sequence of *Hodgkinia*-Ple probe exhibits similarities of 88.24% and 94.12% with the 16S rRNA gene sequences of *Hodgkinia* symbionts in *Auritibicen japonicus* and *Auritibicen bihamatus*, which is related to that 16S rRNA gene sequences of *Hodgkinia* lineages were dissimilar in the probe target sites of these two cicada species (Figure 2, Table 1 and Table S1). We have listed the two other *Hodgkinia* probes (i.e., *Hodgkinia*-Tet and *Hodgkinia*-Mag) in Table 1, which exhibit 100% similarity with the 16S rRNA gene sequences of *Hodgkinia* lineages in related cicadas.

**Figure 3:**
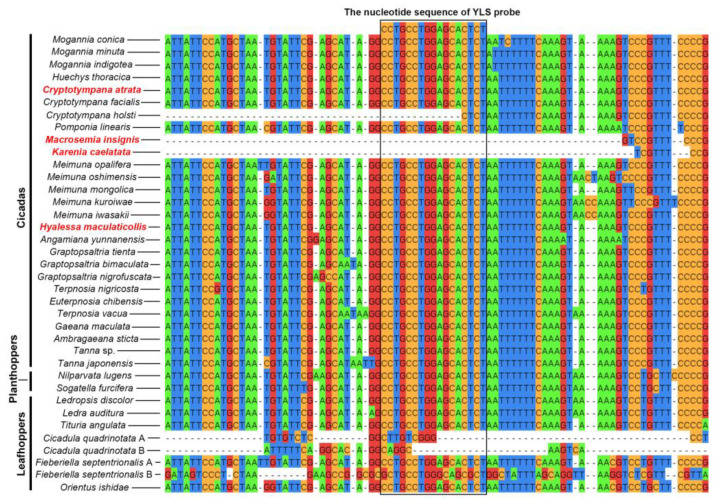
Similarity between the nucleotide sequence of YLS probe and 18S rRNA gene sequences of YLS in cicadas and other representative auchenorrhynchan insects. Different color represents distinct DNA bases. Red font represents the cicada species used in this study.

The nucleotide sequence of the YLS probe used in this study shows 100% similarity with the 18S rRNA gene sequences of YLS symbionts in cicadas (Figure 3, Table 1 and Table S1). The nucleotide sequence of YLS probe exhibits 100% similarity with the 18S rRNA gene sequences of YLS in almost all planthoppers and leafhoppers except for *Fieberiella septentrionalis*, but it shows similarities of 64.71% and 100% with the 18S rRNA gene sequences of YLS in the leafhopper *F. septentrionalis* in which YLS has split into two different lineages (Figure 3, Table 1 and Table S2).

### 2.2. Distribution of Sulcia, Hodgkinia and YLS in Bacteriomes and Fat Bodies of Seven Representative Cicada Species

**Figure 4:**
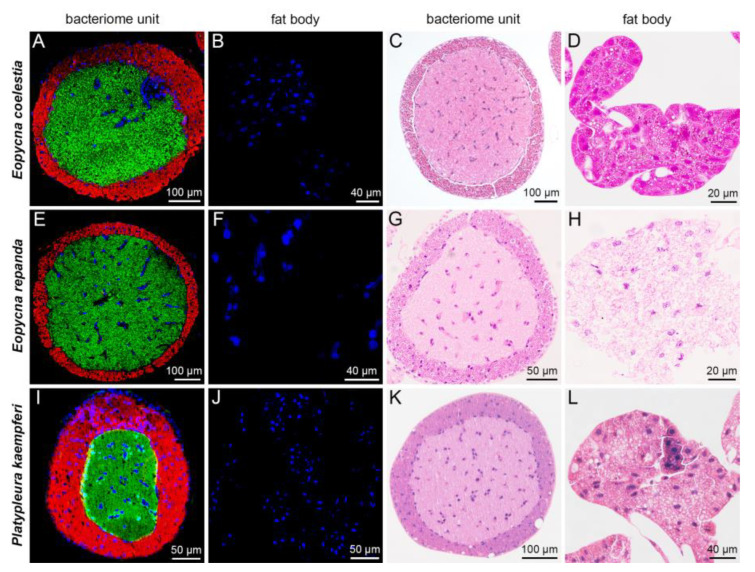
Distribution of *Sulcia* and *Hodgkinia* in the bacteriomes and fat bodies of *Eopycna coelestia*, *Eopycna repanda* and *Platypleura kaempferi*. (**A**–**D**) *Sulcia* in peripheral bacteriocytes and *Hodgkinia* in central bacteriocytes of bacteriomes in *E. coelestia*. (**E**–**H**) *Sulcia* in peripheral bacteriocytes and *Hodgkinia* in central bacteriocytes of bacteriomes in *E. repanda*. (**I**–**L**) *Sulcia* in peripheral bacteriocytes and *Hodgkinia* in central bacteriocytes of bacteriomes in *P. kaempferi*. For fluorescence microscopy, blue, red and green represent nucleus, *Sulcia* and *Hodgkinia*, respectively.

**Figure 5:**
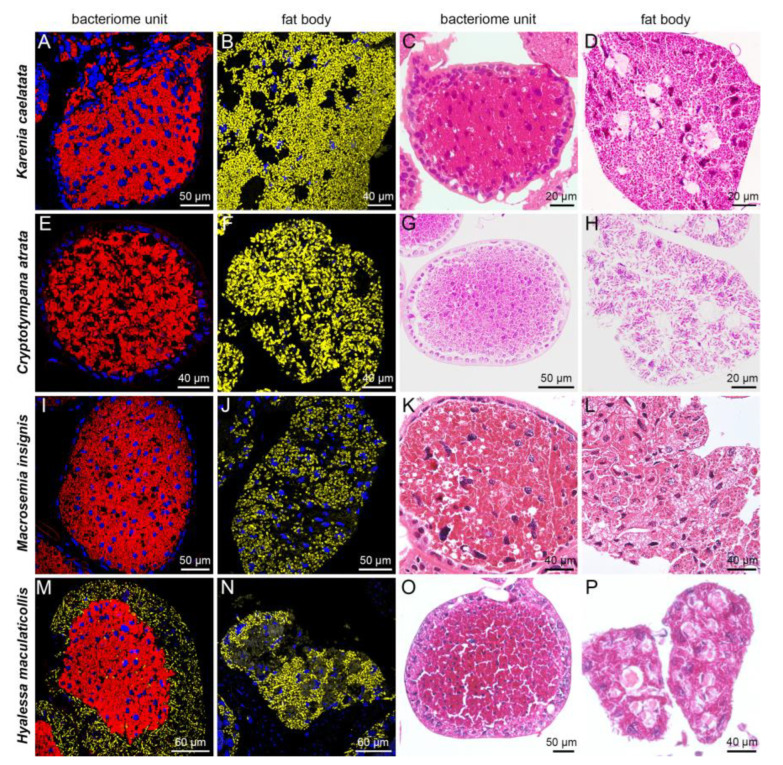
Distribution of *Sulcia* and YLS in the bacteriomes and fat bodies of *Karenia caelatata*, *Cryptotympana atrata*, *Macrosemia insignis* and *Hyalessa maculaticollis*. (**A**–**D**) *Sulcia* in bacteriocytes of bacteriomes and YLS in fat bodies of *K. caelatata*. (**E**–**H**) *Sulcia* in bacteriocytes of bacteriomes and YLS in fat bodies of *C. atrata*. (**I**–**L**) *Sulcia* in bacteriocytes of bacteriomes and YLS in fat bodies of *M. insignis*. (**M**–**P**) *Sulcia* in bacteriocytes of bacteriomes, while YLS in fat bodies and bacteriome sheath of *H. maculaticollis*. For fluorescence microscopy, blue, yellow and red represent nucleus, YLS and *Sulcia*, respectively.

For *E*. *coelestia*, *E*. *repanda* and *P. kaempferi* that harbored *Hodgkinia* and *Sulcia* in the bacteriomes, *Sulcia* was harbored in the peripheral bacteriocytes, whereas *Hodgkinia* occupied the central bacteriocytes of bacteriome units (Figure 4A–L). *Sulcia* was harbored in the bacteriomes of *K*. *caelatata*, *C. atrata* and *M. insignis*, whereas YLS was only present in the fat bodies of these three species (Figure 5A–L). In contrast, *Sulcia* was harbored in the bacteriomes of *H*. *maculaticollis*, and YLS was harbored in both the bacteriome sheath and fat bodies (Figure 5M–P). 

The composition of symbionts harbored in the seven cicada species can be divided into three categories according to the distribution of YLS, *Hodgkinia* and *Sulcia* in the bacteriomes and/or fat bodies. The categories are schematically illustrated (Figure S1) and described as follows. Category 1: *Sulcia* only harbored in the bacteriocytes of bacteriomes, with YLS only harbored in the fat bodies (*K*. *caelatata*, *C. atrata* and *M. insignis*). Category 2: *Sulcia* harbored in the bacteriocytes of bacteriomes, and YLS harbored in both the bacteriome sheath and fat bodies (*H*. *maculaticollis*). Category 3: *Hodgkinia* and *Sulcia* harbored in the bacteriocytes of bacteriomes, with no obligate symbiont harbored in the fat bodies (*E*. *coelestia*, *E*. *repanda* and *P. kaempferi*).

### 2.3. The Improvement of the FISH Signal Intensity of Symbionts by Addition of Unlabeled Helper Sequences and Heat Shock

**Figure 6:**
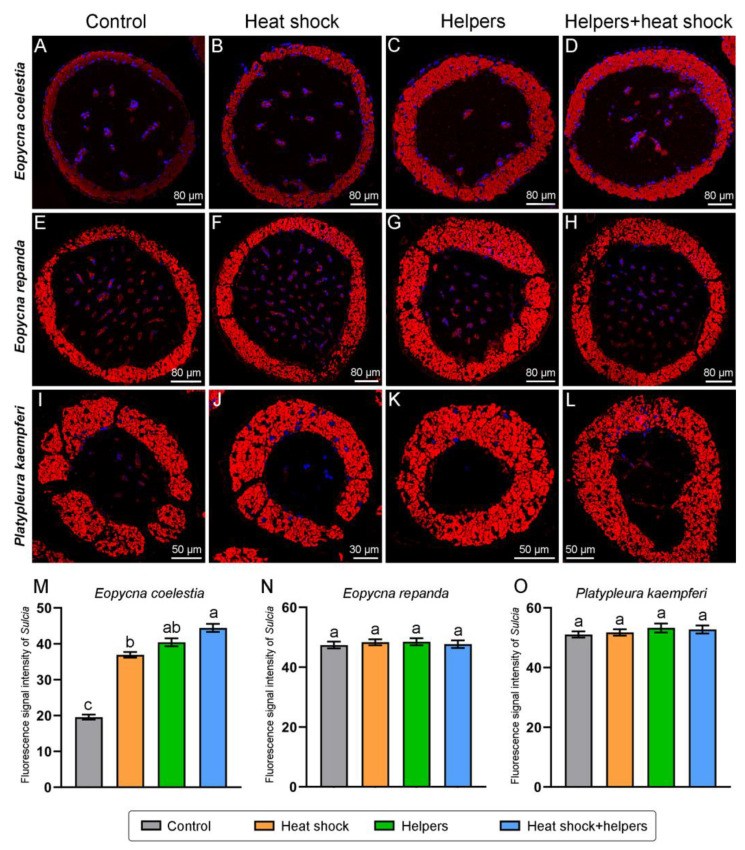
Fluorescent microscopy showing the distribution and FISH signal intensities of *Sulcia* under different treatment groups and the control group in *Eopycna coelestia*, *Eopycna repanda* and *Platypleura kaempferi*. Fluorescent microscopy revealing the distribution and hybridization signal of *Sulcia* in bacteriomes of *E. coelestia* (**A**–**D**), *E. repanda* (**E**–**H**) and *P. kaempferi* (**I**–**L**). Statistical analysis revealing the difference in the hybridization signal intensities of *Sulcia* in *E. coelestia* (**M**), *E. repanda* (**N**) and *P. kaempferi* (**O**), and different letters represent significant differences at the 0.05 level. For fluorescence microscopy, blue and red represent nucleus and *Sulcia*, respectively. Abbreviations: control, without addition of helpers and heat shock; helpers, hybridization buffer containing unlabeled oligonucleotides binding to the adjacent probe target sites; heat shock, addition of 95 °C heat shock for 2 min before hybridization; helpers + heat shock, addition of helpers and heat shock. Error bars represent the standard error in the column graph.

**Figure 7:**
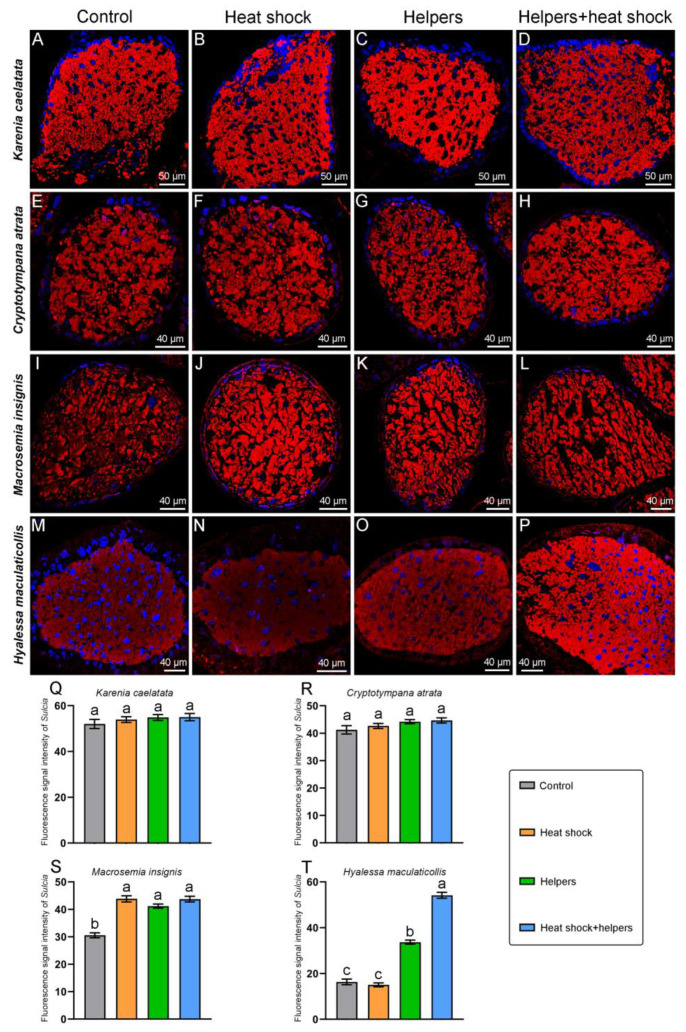
Fluorescent microscopy showing the distribution and FISH signal intensities of *Sulcia* under different treatment groups and the control group in *Karenia caelatata, Cryptotympana atrata, Macrosemia insignis* and *Hyalessa maculaticollis*. Fluorescent microscopy revealing the distribution and hybridization signal of *Sulcia* in the bacteriomes of *K. caelatata* (**A**–**D**), *C. atrata* (**E**–**H**), *M. insignis* (**I**–**L**) and *H. maculaticollis* (**M**–**P**). Statistical analysis revealing the difference in the hybridization signal intensities of *Sulcia* in *K. caelatata* (**Q**), *C. atrata* (**R**), *M. insignis* (**S**) and *H. maculaticollis* (**T**), and different letters represent significant differences at the 0.05 level. For fluorescence microscopy, blue and red represent nucleus and *Sulcia*, respectively. Abbreviations: control, without addition of helpers and heat shock; helpers, hybridization buffer containing unlabeled oligonucleotides binding to the adjacent probe target sites; heat shock, addition of 95 °C heat shock for 2 min before hybridization; helpers + heat shock, addition of helpers and heat shock. Error bars represent the standard error in the column graph.

The fluorescence signal intensity of *Sulcia* in *E*. *repanda*, *P. kaempferi*, *K*. *caelatata* and *C*. *atrata* is generally similar under the control group and three treatment groups (Figure 6 and Figure 7). *Sulcia* of these four cicada species exhibit bright fluorescence signals under the control group, and it also shows strong hybridization signals under the other three treatment groups (viz., the addition of helpers and/or heat shock). The *Sulcia* fluorescence signal intensities of the control group/heat shock group/helpers group/helpers + heat shock group in *E*. *repanda*, *P. kaempferi*, *K*. *caelatata* and *C*. *atrata* are approximately 1:1.02:1.02:1.01, 1:1.01:1.04:1.03, 1:1.04:1.05:1.06, and 1:1.03:1.07:1.08, respectively. For *E*. *coelestia* and *M. insignis*, the fluorescence signal intensity of *Sulcia* can be enhanced by the addition of helpers and/or heat shock when compared with that of the control group (Figure 6 and Figure 7). Although the fluorescence signal of *Sulcia* in *H*. *maculaticollis* can be significantly enhanced by the addition of helpers, *Sulcia* still exhibited quite a weak fluorescence signal under this treatment group (Figure 7). However, *Sulcia* of *H*. *maculaticollis* exhibits the brightest fluorescence signals by the addition of both heat shock and helpers when compared with that of the control group and the other two treatment groups (viz., the addition of only helpers or heat shock) (Figure 7). The *Sulcia* fluorescence signal intensities of the control group/heat shock group/helpers group/helpers + heat shock group in *M. insignis*, *H*. *maculaticollis* and *E*. *coelestia* are approximately 1:1.43:1.35:1.43, 1:0.92:2.07:3.32 and 1:1.89:2.07:2.27, respectively. Therefore, the addition of helpers or heat shock can enhance the fluorescence signal of *Sulcia* in *H*. *maculaticollis, E*. *coelestia* and *M. insignis*, but *Sulcia* exhibited the brightest signals by addition of both heat shock and helpers in these three species (Figure 6 and Figure 7).

**Figure 8:**
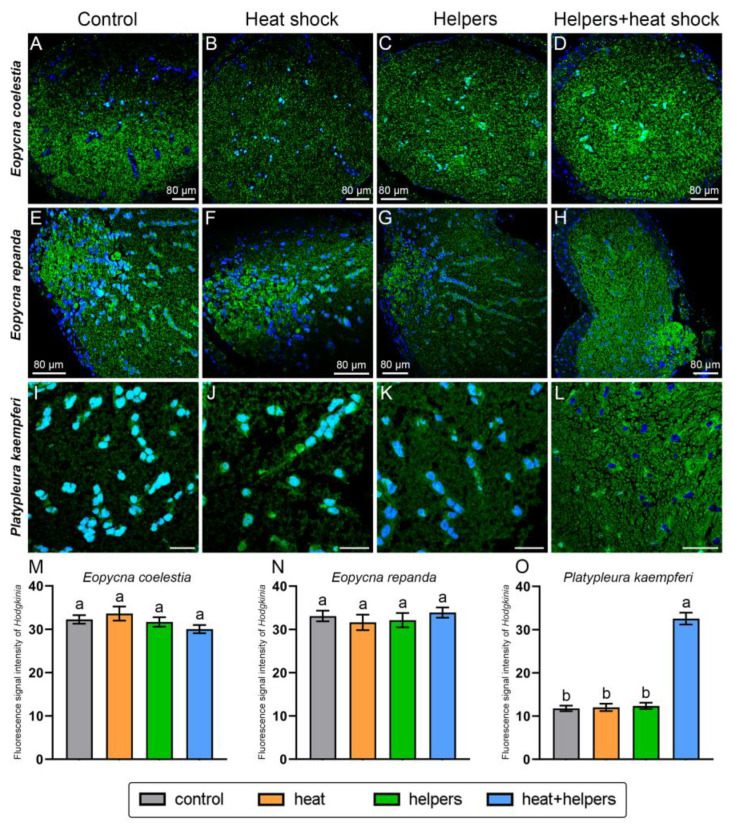
Fluorescent microscopy showing the distribution and FISH signal intensities of *Hodgkinia* under different treatment groups and control group in *Eopycna coelestia*, *Eopycna repanda* and *Platypleura kaempferi*. Fluorescent microscopy revealing the distribution and hybridization signal of *Hodgkinia* in the bacteriomes of *E. coelestia* (**A**–**D**), *E. repanda* (**E**–**H**) and *P. kaempferi* (**I**–**L**). Statistical analysis revealing the difference in the hybridization signal intensities of *Hodgkinia* under three treatment groups and control group in *E. coelestia* (**M**), *E. repanda* (**N**), and *P. kaempferi* (**O**), and different letters represent significant differences at the 0.05 level. For fluorescence microscopy, blue and green represent nucleus and *Hodgkinia*, respectively. Abbreviations: control, without addition of helpers and heat shock; helpers, hybridization buffer containing unlabeled oligonucleotides binding to the adjacent probe target sites; heat shock, addition of 95 °C heat shock for 2 min before hybridization; helpers + heat shock, addition of helpers and heat shock. Error bars represent the standard error in the column graph.

The fluorescence signal intensity of *Hodgkinia* is generally similar under the control group and three treatment groups in *E*. *coelestia* and *E*. *repanda* (Figure 8). *Hodgkinia* of *E*. *coelestia* and *E*. *repanda* show bright fluorescence signals under the control group and also the three treatment groups (viz., the addition of helpers and/or heat shock). The addition of helpers or heat shock cannot significantly enhance the fluorescence signal of *Hodgkinia* in *P. kaempferi* when compared with that of the control group, but the fluorescence signal of *Hodgkinia* can be significantly improved by the addition of both heat shock and helpers (Figure 8). The *Hodgkinia* fluorescence signal intensities of the control group/heat shock group/helpers group/helpers + heat shock group in *E*. *coelestia*, *E*. *repanda* and *P. kaempferi* are approximately 1:1.04:0.98:0.93, 1:0.96:0.97:0.99 and 1:1.02:1.05:2.77, respectively. 

**Figure 9:**
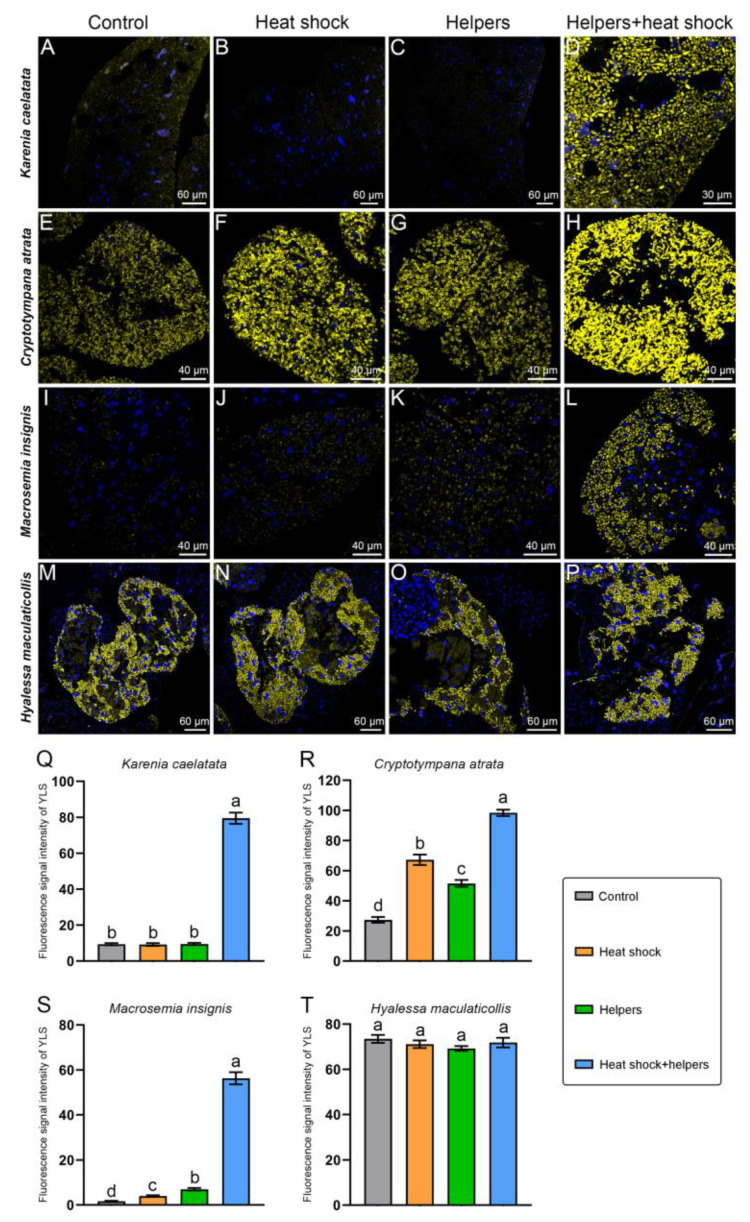
Fluorescent microscopy showing the distribution and FISH signal intensities of YLS under different treatment groups and control group in *Karenia caelatata*, *Cryptotympana atrata*, *Macrosemia insignis* and *Hyalessa maculaticollis*. Fluorescent microscopy revealing the distribution and hybridization signal of YLS in fat bodies of *K. caelatata* (**A**–**D**), *C. atrata* (**E**–**H**), *M. insignis* (**I**–**L**) and *H. maculaticollis* (**M**–**P**). Statistical analysis revealing the difference in the hybridization signal intensities of YLS in *K. caelatata* (**Q**), *C. atrata* (**R**), *M. insignis* (**S**) and *H. maculaticollis* (**T**). Different letters represent significant differences at the 0.05 level. For fluorescence microscopy, blue and yellow represent nucleus and YLS, respectively. Abbreviations: control, without addition of helpers and heat shock; helpers, hybridization buffer containing unlabeled oligonucleotides binding to the adjacent probe target sites; heat shock, addition of 95 °C heat shock for 2 min before hybridization; helpers + heat shock, addition of helpers and heat shock. Error bars represent the standard error in the column graph.

The YLS of *K*. *caelatata* and *M. insignis* exhibited weak fluorescence signals without the addition of heat shock and helpers, whereas it still showed weak hybridization signals by the addition of helpers or heat shock in these two species (Figure 9). The fluorescence signal intensity of YLS can be significantly enhanced by the addition of both heat shock and helpers in *K*. *caelatata* and *M. insignis* when compared with that of the control group and other two treatment groups (viz., the addition of helpers or heat shock) (Figure 9). The fluorescence signal intensity of YLS in *C*. *atrata* can be significantly enhanced by the addition of heat shock and/or helpers when compared with that of the control group (Figure 9). Notably, YLS of *K*. *caelatata, C*. *atrata* and *M. insignis* exhibited the brightest fluorescence signal by the addition of both helpers and heat shock when compared with that of the control group and the other two treatment groups (viz., the addition of helpers or heat shock). YLS of *H*. *maculaticollis* exhibited a bright fluorescence signal under the control groups, which also showed quite a bright fluorescence signal under the three treatment groups (viz., the addition of helpers and/or heat shock). The YLS fluorescence signal intensities of the control group/heat shock group/helpers group/helpers + heat shock group in *K*. *caelatata, C*. *atrata*, *M. insignis* and *H*. *maculaticollis* were approximately 1:0.98:1.01:8.50, 1:2.46:1.88:3.60, 1:2.37:4.20:33.89 and 1:0.97:0.94:0.98, respectively.

### 2.4. The Predicted Secondary Structure of 16S rRNA/18S rRNA Gene Sequence of Symbionts in Seven Cicadas

For the secondary structure of 16S rRNA/18S rRNA gene sequences, a stem refers to consecutive base pairs with no unpaired bases separating them, and a hairpin loop refers to consecutive unpaired bases that are closed by one base pair (Figures S2–S5). The secondary structure of *Sulcia* 16S rRNA gene sequences is morphologically distinct among these seven cicada species, and the secondary structure of the *Sulcia* probe target region is also dissimilar in these cicadas (Figures S2 and S3). Three stems and three hairpin loops were shown in the secondary structure of the *Sulcia* probe target region in *H*. *maculaticollis*, *E*. *coelestia*, *E*. *repanda* and *P. kaempferi* (Figures S2 and S3). A stem and two hairpin loops were shown in the secondary structure of the *Sulcia* probe target region in *K*. *caelatata* and *M. insignis* (Figure S3). Three stems and four hairpin loops were shown in the *Sulcia* probe target region in *C*. *atrata* (Figure S3). The secondary structure of the *Sulcia* probe target region was generally similar among *E*. *coelestia*, *E*. *repanda* and *P. kaempferi*, which was also generally similar between *K*. *caelatata* and *M. insignis* (Figures S2 and S3). However, the secondary structure of the *Sulcia* probe target region was unique in *C*. *atrata* and *H*. *maculaticollis* (Figure S3).

The secondary structure of *Hodgkinia* 16S rRNA gene sequences is morphologically distinct among *E*. *coelestia*, *E*. *repanda* and *P. kaempferi* (Figure S4). Two stems and two hairpin loops were shown in the secondary structure of the *Hodgkinia* probe target region in two representative *Hodgkinia* lineages of *E*. *coelestia.* In contrast, four stems and four hairpin loops were shown in the secondary structure of *Hodgkinia* probe target region in a *Hodgkinia* lineage of *E*. *repanda,* whereas three stems and three hairpin loops were shown in the secondary structure of *Hodgkinia* probe target region in another *Hodgkinia* lineage of this cicada species. Two stems and two hairpin loops were shown in the secondary structure of the *Hodgkinia* probe target region in the two *Hodgkinia* lineages of *P. kaempferi*. Furthermore, the secondary structure of the *Hodgkinia* probe target site was dissimilar among *E*. *coelestia*, *E*. *repanda* and *P. kaempferi*, which was significantly distinct in different *Hodgkinia* lineages within a cicada species (Figure S4).

The secondary structure of YLS 18S rRNA gene sequences is morphologically distinct between *C*. *atrata* and *H*. *maculaticollis* (Figure S5). Four stems and four hairpin loops were illustrated in the secondary structure of the YLS probe target region in *C*. *atrata*. In contrast, four stems and three hairpin loops were shown in the secondary structure of the YLS probe target region in *H*. *maculaticollis* (Figure S5). The secondary structure of the YLS probe target region was dissimilar between these two species.

## 3. Discussion

### 3.1. Sample Preservation and Paraffin Section

The quality of preserved samples is quite important for the success of FISH experiments. Fresh animal samples should be immediately dissected and then preserved in the fixatives, which are closely related to the quality of FISH images. Sample fixation can preserve the integral morphology of the tissue samples and prevent cell lysis from enzymatic digestion. Sample fixation permeabilizes the cell membrane to make the rRNA-targeted oligonucleotide probe more easily diffuse to the intracellular rRNA-targeted site, thereby increasing the accessibility of the probe’s target region [2]. In certain cases, additional permeabilization steps, such as lysozyme treatment, may improve the permeability of cell walls of target microbes (e.g., some fungi) in order to enhance the accessibility of oligonucleotide probes [1]. The commonly used fixatives are ethanol, Carnoy solution and 4% paraformaldehyde for the fixation of animal samples, but there is still no standard protocol for sample fixation [1]. It is recommended to use the 100% ethanol or Carnoy solution for the fixation of fungi, including YLS, which contains an incrassate cell wall [21,23]. We recommend that samples be preserved in the freshly prepared fixatives, and the total volume of samples should be less than 10% of the volume of the fixative.

Paraffin sections are especially crucial for the quality of FISH images. It is important to optimize conditions for the paraffin infiltration, embedding and section. For example, the exposure time for dehydrating the animal tissues by soaking in absolute ethanol mainly depended on the type of specimens. Paraffin block was generally cut into 4 μm thick sections, which can be used for histological and fluorescence microscopy. We recommend consulting a technician who has rich experience in the paraffin sections when cutting tissue sections with a microtome for the first time. Future studies are required to explore whether other methods, including whole-mount in situ hybridization of dissected tissues, can improve the fluorescence signal intensities of related symbionts. 

### 3.2. The Selection of rRNA-Targeted Probes for Hybridization Experiments

In contrast to polynucleotide probes, oligonucleotide probes can improve the precision in discriminating similar target sequences of microbes at the genus or species level, and they also easily access the probe target site of rRNA molecules [2]. Currently, the oligonucleotide probe is the most suitable probe used for FISH experiments. A fluorescently labeled oligonucleotide probe is generally 15–25 bp in length, which can be generated on an automated synthesizer [1]. We recommend using the published oligonucleotide probes, which have been successfully used to detect related microorganisms and obtain high-quality FISH images under confocal laser scanning microscopy. Furthermore, it is extremely important to check the identities of nucleotide sequence between oligonucleotide probe and 16S rRNA/18S rRNA gene sequence of microbe(s) with the Nucleotide-BLAST algorithm, aiming to determine whether there are some mismatches between probe sequence and probe target sequence.

In the present study, we revealed that the nucleotide sequence of the *Sulcia* probe shows 100% similarity with the 16S rRNA gene sequences of *Sulcia* symbionts in all cicadas (Figure 1 and Table S1). The *Sulcia* probe sequence exhibits 100% similarity with the 16S rRNA gene sequences of *Sulcia* symbionts in some leafhoppers and planthoppers, whereas it shows 95% similarity with the 16S rRNA gene sequences of *Sulcia* in some leafhoppers, planthoppers and treehoppers (Figure 1 and Table S2). We conclude that the *Sulcia* probe used in this study is suitable for all cicadas and some species of Auchenorrhyncha, but the similarity between the nucleotide sequence of the *Sulcia* probe with the 16S rRNA gene sequences of *Sulcia* symbionts in some other auchenorrhychans can be increased if a more suitable probe of *Sulcia* was selected.

We also revealed that the nucleotide sequence of the *Hodgkinia*-Ple probe shows 100% similarity with the 16S rRNA gene sequences of *Hodgkinia* symbionts in cicadas of the tribe Platypleurini, including *P. kaempferi*, *E*. *coelestia* and *E*. *repanda*, whereas it shows a relatively low similarity with the 16S rRNA gene sequences of *Hodgkinia* symbionts in some other cicada species such as *T. undata* (Figure 2 and Table S1). These results suggest that some oligonucleotide probes can be successfully used to obtain bright FISH images in some species (e.g., *E*. *coelestia*), but they may not be the most suitable probes in other species (e.g., *T. undata*). We conclude that the *Hodgkinia*-Ple probe is the most suitable probe for the detection of *Hodgkinia* symbionts in species of Platypleurini (e.g., *P. kaempferi*, *E*. *coelestia* and *E*. *repanda*), but it may be unsuitable for other species such as species of *Auritibicen*, *Magicicada*, *Tettigetta*, *Kosemia* and *Tettigades.*

We revealed that the nucleotide sequence of YLS probe shows 100% similarity with the 18S rRNA gene sequences of YLS symbionts in cicadas (Figure 3 and Table S1), and the nucleotide sequence of YLS probe exhibits 100% similarity with the 18S rRNA gene sequences of YLS symbionts in almost all planthoppers and leafhoppers, but it shows 64.71% and 100% similarity in the leafhopper *F. septentrionalis* in which YLS split into two lineages (Figure 3 and Table S2). These results indicate that the YLS probe used in this study is suitable for the detection of YLS in almost all cicadas, planthoppers and leafhoppers, but it is unsuitable for the detection of YLS in some auchenorrhynchan insects that harbor complex YLS lineages.

An oligonucleotide probe is generally labeled at the 5′ end of the probe sequence with fluorochromes such as CY3 and CY5. Several types of fluorochromes that have different excitation and emission parameters are commercially available, thereby simultaneously detecting two or more microbes using oligonucleotide probes labeled with different fluorochromes under a confocal laser scanning microscopy. These oligonucleotide probes could be stored in the dark for several months at −20 °C or −80 °C, and the success of FISH experiments is closely related to the storage conditions of probes. We recommend increasing the final concentration of probes used in the hybridization buffer when oligonucleotide probes have been stored for more than one year. Alternatively, it would be better to use a newly synthesized probe in FISH experiments. Microscopy observations should be performed as soon as possible after mounting the hybridization slides with the microscope glass coverslips. Additionally, we can search for some published oligonucleotide probes or design new probes under the publically available probe database (e.g., probeBase) following the guidelines [9]. Probes should be designed as previously described, including the 15–25 bases in length, 50–70% GC content and 50–60 °C Tm [1]. However, it is necessary to examine the specificity of a newly designed probe by conducting diagnostic PCR using an unlabeled oligonucleotide and a universal forward primer such as 16SA1 for the bacterial 16S rRNA gene [1]. 

### 3.3. The Enhancement of Fluorescence Signal Intensity in Symbionts by Addition of Unlabeled Helper Sequences and Heat Shock 

Over the past three decades, FISH with rRNA-targeted oligonucleotide probe has become a widely used method in the detection of microorganisms in complex samples, including animal tissues and clinical specimens [1,2]. However, the FISH technique still suffers from some limitations, including unavailable probe binding sites of 16 rRNA or 18 rRNA, low cellular ribosome content and inaccessibility of the probe binding sites resulting in the weak or even absence of fluorescence signal intensity of targeted microbes, which may eventually lead to underestimation or misunderstanding of the density and distribution of related microbes [2].

It has been revealed that unlabeled oligonucleotides (helpers) binding to the adjacent probe target sites can open inaccessible rRNA regions for FISH with oligonucleotide probes, thereby increasing the hybridization signal intensities of the targeted microbe(s) [2,26,27]. Previous studies revealed that helpers binding adjacent to the probe target site can increase weak probe hybridization signals in *Escherichia coli* based on the results of flow cytometric analysis [26,27]. A previous study reported that the addition of helpers and heat shock greatly enhanced the FISH signal intensity of YLS in cicada *Tanna* sp. and *K*. *caelatata,* whereas the FISH signal intensity of *Sulcia* was generally similar between the control group and treatment group (viz., the addition of both helpers and heat shock) [23]. However, it is still unclear whether the addition of helpers and/or heat shock can improve the FISH signal intensity of YLS, *Sulcia* and *Hodgkinia* in other cicada species. In the present study, we revealed that the fluorescence signal intensity of *Sulcia* can be significantly enhanced by the addition of heat shock and/or helpers in *M. insignis*, *H*. *maculaticollis* and *E*. *coelestia*. *Sulcia* of these three species exhibited the brightest signals by the addition of both heat shock and helpers, although the fluorescence signal of *Sulcia* can be enhanced by the addition of helpers or heat shock (Figure 6 and Figure 7). The addition of helpers or heat shock cannot enhance the fluorescence signal of *Hodgkinia* in *P. kaempferi* when compared with that of the control group, but the fluorescence signal of *Hodgkinia* can be significantly improved by the addition of both heat shock and helpers (Figure 8). The fluorescence signal intensity of YLS can be significantly enhanced by the addition of heat shock and helpers in *K*. *caelatata*, *C. atrata* and *M. insignis*. Notably, the YLS of *K*. *caelatata, C*. *atrata* and *M. Insignis* exhibited the brightest fluorescence signal by the addition of both helpers and heat shock (Figure 9). Our results confirm that the fluorescence signal of *Sulcia*, *Hodgkinia* and YLS exhibited weak intensity without the addition of unlabeled oligonucleotides (helpers) and heat shock in some cicadas; however, it can be significantly improved by the addition of both helpers and heat shock. The results support the previous conclusion that modification by the addition of helpers and/or heat shock can enhance the FISH signal intensity of symbionts in insects and other animals [23,28,29]. These results suggest a potential mechanism that heat shock denaturing rRNA and proteins of related microbe(s) together with unlabeled helpers binding to the denatured rRNA prevent the re-establishment of the native secondary structure of rRNA and, therefore, suitable probe(s) can more easily access to the probe’s target sites of rRNA. Generally, the modified FISH technique by addition of both helpers and heat shock can significantly enhance the fluorescence signals of related symbionts, which exhibited quite low hybridization signals without the addition of them. Therefore, we recommend improving the fluorescence signals of related symbionts using a modified FISH technique through the addition of both helpers and heat shock, especially for symbionts that exhibit quite weak or even no hybridization signals using a standard FISH method. However, we cannot rule out the possibility that there may be some tightly closed regions on which the addition of helpers and heat shock fails to open the probe’s target sites.

The *Sulcia* fluorescence signal intensities of the control group/heat shock group/Helpers group/helpers + heat shock group in *M. insignis*, *H*. *maculaticollis* and *E*. *coelestia* were approximately 1:1.43:1.35:1.43, 1:0.92:2.07:3.32 and 1:1.89:2.07:2.27, respectively (Figure 6 and Figure 7). The YLS fluorescence signal intensities of the control group/heat shock group/helpers group/helpers + heat shock group in *K*. *caelatata, C*. *atrata* and *M. insignis* were approximately 1:0.98:1.01:8.50, 1:2.46:1.88:3.60 and 1:2.37:4.20:33.89, respectively. (Figure 9). We revealed that a stem and two hairpin loops were shown in the secondary structure of the *Sulcia* probe target region in *K*. *caelatata* and *M. insignis.* Three stems and four hairpin loops were shown in the target region of *Sulcia* probe in *C*. *atrata.* Three stems and three hairpin loops were shown in the secondary structure of *Sulcia* probe target region in *H*. *maculaticollis*, *E*. *coelestia*, *P. kaempferi* and *E*. *repanda* (Figures S2 and S3). Additionally, four stems and four hairpin loops were illustrated in the secondary structure of the YLS probe target region in *C*. *atrata*. In contrast, four stems and three hairpin loops were shown in the secondary structure of the YLS probe target region in *H*. *maculaticollis*. The secondary structure of the YLS probe target region was dissimilar between these two species (Figure S5). Presumably, different efficiencies of FISH signal intensity by the addition of helpers may be closely related to the differences in the secondary structure of the 16 rRNA/18 rRNA of symbionts in cicadas, which includes rRNA–rRNA or rRNA–protein interaction at some specific regions.

In summary, we revealed that *Sulcia* of *K*. *caelatata*, *C*. *atrata*, *P. kaempferi* and *E*. *repanda* exhibited bright fluorescence signals under the control group, and it also showed strong hybridization signals under the other three treatment groups (viz., the addition of helpers and/or heat shock) (Figure 6 and Figure 7). Similarly, *Sulcia/Hodgkinia* of *E*. *coelestia* and *E*. *repanda* showed bright fluorescence signals under the control groups and other three treatment groups (viz., the addition of helpers and/or heat shock) (Figure 6 and Figure 8). We hypothesize that the accessibility of the probe’s target region in some cicadas (e.g., *E*. *coelestia* and *E*. *repanda*) keep open even if without the addition of helpers and heat shock, and the oligonucleotide probe can easily access the probe’s target region of 16 rRNA/18 rRNA of symbionts. However, it is still necessary to search for the optimal conditions of each microbe, and the combination of these seems to be a good choice for the detection of two or more microbes under confocal microscopy in a FISH experiment.

## 4. Materials and Methods

### 4.1. Sample Collection and Dissection

The adults of seven cicada species were collected in the field during the cicada emergence period between 2019 and 2022. Detailed information on the seven cicadas has been listed in Table S3. The dissection of bacteriomes and fat bodies was performed as previously described [12]. Briefly, the bacteriomes and fat bodies were immediately preserved in 100% ethanol at a 4 °C refrigerator for histological and fluorescence microscopy.

### 4.2. Compared the Similarity between rRNA-Targeted Oligonucleotide Probe and 16S rRNA/18S rRNA Gene Sequence of Targeted Microbes

We checked the identities of the rRNA-targeted oligonucleotide probe and 16S rRNA/18S rRNA gene sequences of targeted microbe(s) (i.e., *Sulcia*, *Hodgkinia* and YLS) by the alignment of probe sequence and rRNA-targeted gene sequence(s) in the BLAST searches under the Nucleotide-BLAST program. The NCBI accession numbers of the 16S rRNA/18S rRNA gene sequence of symbionts in cicadas and other insects have been listed in the Supplementary Materials (Tables S4 and S5). Multiple alignments of the 16S rRNA/18S rRNA gene sequences of the symbionts (*Sulcia*, *Hodgkinia* and YLS) and related probe sequences were conducted using the MEGA version 11.0 as previously described [23]. The multiple alignment of sequences was exported as a FASTA file and then imported into the Jalview version 2.8.2 [30]. Jalview version 2.8.2 provides a choice for each of the color schemes available in the “Colour” menu, and alignment views can be exported in a range of ways via the “Export” submenu [30]. Sequence alignment was exported as Portable Network Graphic (PNG) raster images when preparing figures for publication.

### 4.3. Fluorescence In Situ Hybridization

The bacteriomes and fat bodies were dehydrated in a graded ethanol series (75% for 4 h, 85% for 2 h, 90% for 2 h, 95% for 1 h and 100% for 30 min twice), cleared four times in xylene for 2 h, and finally embedded with melted paraffin. Paraffin blocks were sectioned to 4 μm. Thin sections were used for fluorescence or histological microscopy. Sections used for histological microscopy were stained with hematoxylin and eosin, and the detailed protocol was the same as previously described [23]. Histological sections were finally mounted with neutral balsam (Sigma, Saint Louis, MI, USA) and photographed under a light microscope BX53M (Olympus, Tokyo, Japan). 

The FISH assay was performed as previously described with some modifications [18,19,25]. We provided a schematic representation showing the oligonucleotide probe and unlabeled helpers binding to the rRNA of related symbionts (e.g., YLS, *Sulcia* and *Hodgkinia*) in cicadas (Figure S6). The detailed components of the hybridization buffer were listed in Supplementary Materials (Tables S6–S8). The sequences of all probes and helper sequences used in the present study were listed in Table 1 and Table S9, respectively. The detailed protocol for the hybridization was the same as previously described [23].

We compared the fluorescence signal intensities of YLS, *Sulcia* and *Hodgkinia* based on different treatments, aiming to explore whether the hybridization signals of these symbionts can be significantly enhanced by the addition of unlabeled helper sequences and/or heat shock (Tables S10–S12). The control group represents a hybridization buffer with no containing helpers (Table S7) and no addition of 95 °C heat shock before hybridization. The heat shock group represents the addition of 95 °C heat shock for 2 min before hybridization, but no addition of helpers in the hybridization buffer (Table S7). The helpers group represents the addition of helpers in the hybridization buffer (Table S8), but no addition of 95 °C heat shock for 2 min before hybridization. The heat shock + helpers group represents the addition of both helpers (Table S8) and heat shock. Briefly, each slide was carried out in a final volume of 50 μL hybridization buffer. Hybridization was conducted in a 37 °C humidified chamber overnight [28,29]. In addition, the negative control was performed using no probe staining to check the specificity of hybridization.

Slides were mounted in a commercially available antifade medium and immediately observed and imaged under an Olympus FV 3000 IX inverted laser scanning confocal microscope with a 20× air lens or 40× oil-immersion lens (Olympus, Tokyo, Japan). The image was acquired using the same parameters for the same type of symbiont of the same cicada species. Fluorescence signal intensities of hybridization sections were measured using the Image J version 1.5.3 software. For each treatment, we generally measured approximately 30 distinctly symbiont-harbored areas of the hybridization sections of related symbionts. The normality of data of different groups was initially examined using the Shapiro–Wilk test using SPSS version 27 and R 3.5.2 software (Tables S13–S15). We performed the nonparametric test (Kruskal–Wallis test) to determine the differences in the fluorescence signal intensities of symbionts under the control group and different treatment groups using SPSS version 27 software. Additionally, fluorescence signal intensities of the control group and three treatment groups were compared using the Kruskal–Wallis test for pairwise comparisons.

### 4.4. The Prediction of Secondary Structure of 16S rRNA/18S rRNA Gene Sequence of Symbionts in Six Cicadas

The 16 rRNA/18 rRNA gene sequences of symbionts (i.e., YLS, *Sulcia* and *Hodgkinia*) were predicted using Geneious Primer 2023.2 software according to the minimum free energy (MFE) methods, which is mainly based on the assumption that a DNA molecule folds into the MFE structure among all possible structures [8]. The predicted secondary structure was illustrated in the planar structure (DNA backbone). A DNA strand comprises four different bases, including guanine (G), adenine (A), thymine (T) and cytosine (C), and one base can connect to another base through the DNA backbone. A stem refers to consecutive base pairs with no unpaired bases separating them. In contrast, a hairpin loop refers to consecutive unpaired bases that are closed by one base pair [8].

## 5. Conclusions

We initially revealed that the probes of *Candidatus* Sulcia muelleri (*Sulcia*) and the yeast-like fungal symbiont (YLS) are suitable for detection of these symbionts in all cicadas and other insects of Auchenorrhyncha, whereas the probe of *Candidatus* Hodgkinia cicadicola (*Hodgkinia*) is only suitable for detection of *Hodgkinia* in a few cicada species. The fluorescence signal of *Sulcia, Hodgkinia* and YLS exhibited weak intensity without the addition of helpers and heat shock in some cicadas, which can be significantly improved by the addition of both helpers and heat shock. Our results provide new information for the improvement of hybridization signal intensities of microbes in the FISH experiments with modifications.

## Figures and Tables

**Table 1:** Probes used for fluorescence in situ hybridization of *Sulcia* and YLS of two cicada species.

Probe Name	Suitable for Host Cicadas	Primer Sequence (5′–3′)	References
*Sulcia*	All species	CCACACATTCCAGTTACTCC	[18]
YLS	All species	CCTGCCTGGAGCACTCT	[21]
*Hodgkinia*-Ple	*Eopycna repanda*, *Eopycna coelestia*, *Platypleura kaempferi*	CCAGT GTGGC TGTCC GT	This study
*Hodgkinia*-Tet	*Tettigades undata*, *Diceroprocta semicincta*	CCAAT GTGGC TGACC GT	[25]
*Hodgkinia*-Mag	*Magicicada septendecim*	CCAAT GTGGC TGTYC RT	[19]

## Data Availability

All sequences obtained in this study have been deposited in the NCBI nucleotide database.

## Supplementary Materials

The following supporting information can be downloaded at: https://www.mdpi.com/article/10.3390/ijms242115838/s1.
