# Supplementary material for: The Improvement of Fluorescence In Situ Hybridization Technique Based on Explorations of Symbionts in Cicadas
Source: Int J Mol Sci. 2023 Oct 31;24(21):15838. doi: 10.3390/ijms242115838 (PMC10650757; doi:10.3390/ijms242115838)
Supplement: Supplementary file 1 [file ijms-24-15838-s001.zip › ijms-2661427-supplementary.pdf]

Supplemental materials

Table S1 The sequence similarity between the 16S rRNA/18S rRNA gene sequences and related oligonucleotide probes in cicadas

Cicada species	*Sulcia* probe [a]	*Hodgkinia* probe [b]	YLS probe [c]
Subpsaltria yangi	100%	–	\
Tettigades undata	100%	88.24%	–
Kosemia yezoensis	100%	82.35%	–
Tettigetta sp.	100%	82.35%	–
Huechys thoracica	100%	–	100%
Muda kuroiwae	100%	\	–
Katoa paucispina	100%	\	–
Magicicada septendecim	100%	88.24%	–
Magicicada tredecim	100%	88.24%	–
Mogannia conica	100%	–	100%
Mogannia indigotea	100%	–	100%
Mogannia cyanea	100%	–	\
Mogannia minuta	100%	–	100%
Platypleura kaempferi	100%	100%	–
Eopycna coelestia	100%	100%	–
Eopycna repanda	100%	100%	–
Auritibicen japonicus	100%	88.24% and 94.12%	–
Auritibicen bihamatus	100%	88.24% and 94.12%	–
Auritibicen jai	100%	\	–
Cryptotympana holsti	100%	–	\
Cryptotympana atrata	100%	–	100%
Cryptotympana facialis	100%	–	100%
Pomponia linearis	100%	–	100%
Dundubia hainanensis	100%	–	\
Karenia caelatata	100%	–	\
Macrosemia umbrata	100%	–	\
Macrosemia insignis	100%	–	\
Meimuna iwasakii	100%	–	100%
Meimuna kuroiwae	100%	–	100%
Meimuna mongolica	100%	–	100%
Meimuna oshimensis	100%	–	100%
Meimuna opalifera	100%	–	100%

Hyalessa maculaticollis	100%	–	100%
Graptopsaltria tienta	100%	–	100%
Graptopsaltria nigrofuscata	100%	–	100%
Graptopsaltria bimaculata	100%	–	100%
Purana gigas	100%	–	\
Terpnosia nigricosta	100%	–	100%
Euterpnosia chibensis	100%	–	100%
Terpnosia vacua	100%	–	100%
Gaeana maculata	100%	–	100%
Ambragaeana sticta	100%	–	100%
Tanna sp.	100%	–	100%
Tanna japonensis	100%	–	100%

Table S2 The sequence similarity between the 16S rRNA/18S rRNA gene sequences and related oligonucleotide probes in other auchenorrhynchan insects

Cicada species	*Sulcia* probe [a]	YLS probe [b]
Nephotettix cincticeps	100%	
Macrosteles sexnotatus	100%	
Pamplona spatulate	95%	
Cicadella viridis	95%	
Cuerna striata	95%	
Macrosteles laevis	100%	
Cicadula quadrinotata	100%	\
Fieberiella septentrionalis	100%	64.71% and 100%
Graphocraerus ventralis	100%	
Orientus ishidae	100%	100%
Ledropsis discolor	95%	100%
Ledra auditura	\	100%
Tituria angulata	\	100%
Publilia modesta	95%	
Philya ferruginosa	95%	
Platycotis vittate	95%	
Centrotus cornutus	95%	
Gargara genistae	95%	
Hyalesthes scotti	100%	
Oliarus filicicola	100%	
Aphypia longipennis	100%	
Ommatidiotus dissimilis	100%	
Nilparvatata lugens	\	100%
Sogatella furcifera	\	100%

Table S3 The collection information of cicada samples used in this study

Species	Individuals	Locality	Collection date
Karenia caelatata	28	Ningshan County, Shaanxi Province	middle August of 2016-2021
Hyalessa maculaticollis	26	Ningshan County, Shaanxi Province	late July of 2020-2021
Cryptotympana atrata	25	Ningshan County, Shaanxi Province	late August of 2020-2021
Macrosemia insignis	18	Puer City, Yunnan Province	early August of 2021-2022
Eopycna coelestia	21	Tongjiang County, Sichuan Province	middle April of 2020-2021
Eopycna repanda	36	Meixian County, Shannxi Province	middle August of 2021-2022
Platypleura kaempferi	16	Ningshan County, Shaanxi Province	middle July of 2021-2022

Table S4 The accession numbers of the 16S rRNA/18S rRNA gene sequences of symbionts in cicadas under NCBI nucleotide database

Cicada species	*Sulcia*	*Hodgkinia*	YLS
Subpsaltria yangi	MT533418	–	\
Tettigades undata	CP007234	CP007232–CP007233	–
Kosemia yezoensis	LC370763	LC370599–LC370601, LC370604–LC370606, LC370611–LC370612	–
Tettigetta sp.	MT533444	OP851564–OP851575	–
Huechys thoracica	MT533445	–	MT537692
Muda kuroiwae	LC370764	\	–
Katoa paucispina	MT533439	\	–
Magicicada septendecim	DQ066625	KR607377	–
Magicicada tredecim	CP010828	KR607416	–
Mogannia conica	MT533435	–	MT537690
Mogannia indigotea	MT533437	–	MT537686
Mogannia cyanea	MT533438	–	\
Mogannia minuta	LC370590	–	LC370999
Platypleura kaempferi	MT533432	LC370452–LC370453, LC370456–LC370458, LC370461, LC370463, LC370465, LC370467, LC370470–LC370472, LC370768–LC370771	–
Eopycna coelestia	OP852135	OP851576–OP851583	–
Eopycna repanda	MK770419	MK926459–MK926463	–
Auritibicen japonicus	LC370637	LC370531, LC370534, LC370538, LC370540, LC370541, LC370543, LC370545, LC370547	–
Auritibicen bihamatus	LC370631	LC370505–LC370516	–
Auritibicen jai	MT533428	\	–
Cryptotympana holsti	MT533424	–	MT537699
Cryptotympana atrata	MT533421	–	MT537669
Cryptotympana facialis	LC370653	–	LC370808
Pomponia linearis	MT533387	–	MT537636
Dundubia hainanensis	MT533373	–	\
Karenia caelatata	MT533370	–	OQ222170

Macrosemia umbrata	MT533363	–	\
Macrosemia insignis	OR726129	–	\
Meimuna iwasakii	LC370740	–	LC370979
Meimuna kuroiwae	LC370737	–	LC370966
Meimuna mongolica	MT533379	–	MT537654
Meimuna oshimensis	LC370728	–	LC370946
Meimuna opalifera	LC370576	–	MT537653
Hyalessa maculaticollis	MT533390	–	MT537645
Graptopsaltria tienta	MT533411	–	MT537664
Graptopsaltria nigrofuscata	LC370686	–	LC370839
Graptopsaltria bimaculata	LC370690	–	LC370850
Purana gigas	MT533396	–	\
Terpnosia nigricosta	LC370697	–	LC370879
Euterpnosia chibensis	LC370698	–	LC370888
Terpnosia vacua	LC370693	–	LC370866
Gaeana maculata	MT533404	–	MT537688
Ambragaeana sticta	MT533402	–	MT537696
Tanna sp.	OQ167978	–	OQ222171
Tanna japonensis	LC370573	–	LC370916

Table S5 The accession numbers of the 16S rRNA/18S rRNA gene sequences of symbionts in other auchenorrhynchan insects under NCBI nucleotide database

**Cicada species**	***Sulcia***	**YLS**
Nephotettix cincticeps	CP016223	
Macrosteles sexnotatus	AB795337	
Pamplona spatulate	AY676908	
Cicadella viridis	AY676915	
Cuerna striata	AY676922	
Macrosteles laevis	KR337981	
Cicadula quadrinotata	KY923021	KY923025, KY923026
Fieberiella septentrionalis	KY923022	KY923027, KY923028
Graphocraerus ventralis	KY923023	
Orientus ishidae	KY923024	KY923029
Ledropsis discolor	LC108769	LC108763
Ledra auditura	\	LC108757
Tituria angulata	\	LC108765
Publilia modesta	DQ066641	
Philya ferruginosa	DQ066642	
Platycotis vittate	DQ066643	
Centrotus cornutus	MN082139	
Gargara genistae	MN082143	
Hyalesthes scotti	FN428790	
Oliarus filicicola	CP110506	
Aphypia longipennis	JQ982613	
Ommatidiotus dissimilis	MG515263	
Nilparvatata lugens	\	AF267233
Sogatella furcifera	\	JF773150

Table S6 The hybridization solution containing helper sequences used in this study

**Hybridization solution**	**Final concentration**	**Volume** (100 μl)
25% dextran sulfate	10 %	40 μl
10% bovine serum albumin	0.25 %	2.5 μl
20×SSC	2.5 X	12.5 μl
ssDNA (200 ng/ml)	10 ng/μl	5 μl
Sulcia Probe (2 μM)	200 nM	10 μl
L-helper (100 μM)	2 μM	2 μl
R-helper (100 μM)	2 μM	2 μl
YLS/*Hodgkinia* Probe (2 μM)	200 nM	10 μl
L-helper (100 μM)	2 μM	2 μl
R-helper (100 μM)	2 μM	2 μl
diH_2O	/	12 μl

Table S7 The hybridization solution not containing helper sequences used in this study

**Hybridization solution**	**Final concentration**	**Volume** (100 μl)
25% dextran sulfate	10 %	40 μl
10% bovine serum albumin	0.25 %	2.5 μl
20×SSC	2.5 X	12.5 μl
ssDNA (200 ng/ml)	10 ng/μl	5 μl
Probe (2 μM)	200 nM	10 μl
diH_2O	/	30 μl

Table S8 The hybridization solution containing helper sequences used in this study

**Hybridization solution**	**Final concentration**	**Volume** (100 μl)
25% dextran sulfate	10 %	40 μl
10% bovine serum albumin	0.25 %	2.5 μl
20×SSC	2.5 X	12.5 μl
ssDNA (200 ng/ml)	10 ng/μl	5 μl
Probe (2 μM)	200 nM	10 μl
L-helper (100 μM)	2 μM	2 μl
R-helper (100 μM)	2 μM	2 μl
diH_2O	/	26 μl

Table S9 Helper sequences used for fluorescence in situ hybridization of *Sulcia*, YLS and *Hodgkinia* in cicadas

**Probe name**	**Primer sequence (5'–3')**	**References**
Sulcia-Lhelper	GTTCTGTGTGATCTCTATGCATTTCACCGCT	18
Sulcia-Rhelper	CCTCACTCTAGTTTATCAGTATCAATAGCACTT	
YLS-Lhelper	CTAATGTATTCGAGCAT	23
YLS-Rhelper	TTTTTCAAAGTAAAAGTCCCGT	
Hodgkinia-Lhelper	CGGGRGTCTGGGCCTTGTTCCA	This study
Hodgkinia-Rhelper	TCCCAGACCAGCTATAGATCGT	

Table S10 The sample information for comparison of *Sulcia* signal intensity in control group and three treatment groups in seven cicada species

**Species**	Replications	Number of bacteriome units observed			
		Control group	Heat shock group	Helper group	Heat + Helpers group
Karenia caelatata	10	32	36	33	40
Hyalessa maculaticollis	10	31	30	30	35
Cryptotympana atrata	10	39	45	42	46
Macrosemia insignis	10	30	35	32	40
Eopycna coelestia	10	31	38	46	41
Eopycna repanda	10	40	42	47	43
Platypleura kaempferi	10	36	34	39	36

Table S11 The sample information for comparison of *Hodgkinia* signal intensity in control group and three treatment groups in three cicada species

**Species**	Replications	Number of bacteriome units observed			
		Control group	Heat shock group	Helper group	Heat + Helpers group
Eopycna coelestia	10	33	31	38	36
Eopycna repanda	10	31	31	37	33
Platypleura kaempferi	10	35	32	36	38

Table S12 The sample information for comparison of YLS signal intensity in control group and three treatment groups in four cicada species

**Species**	Replications	Number of fat bodies observed			
		Control group	Heat shock group	Helper group	Heat + Helpers group
Karenia caelatata	10	32	36	33	40
Hyalessa maculaticollis	10	31	35	30	35
Cryptotympana atrata	10	37	30	33	42
Macrosemia insignis	10	31	34	32	36

Table S13 The normality tests of *Sulcia* signal intensity data in control group and three treatment groups of seven cicada species

**Species**	Control group	Heat shock group	Helper group	Heat + Helpers group
Karenia caelatata	0.669	0.028	0.634	0.471
Hyalessa maculaticollis	0.083	0.016	0.517	0.278
Cryptotympana atrata	0.137	0.636	0.346	0.043
Macrosemia insignis	0.554	0.855	0.445	0.048
Eopycna coelestia	0.010	0.516	0.851	0.289
Eopycna repanda	0.532	0.775	0.049	0.582
Platypleura kaempferi	0.378	0.006	0.023	0.036

Note: The value more than 0.5 represents that the data obtained from the group is normal.

Table S14 The normality tests of *Hodgkinia* signal intensity data in control group and three treatment groups of three cicada species

**Species**	Control group	Heat shock group	Helper group	Heat + Helpers group
Eopycna coelestia	0.036	0.036	0.005	0.080
Eopycna repanda	0.046	0.258	0.260	0.584
Platypleura kaempferi	0.731	0.066	0.120	0.011

Note: The value more than 0.5 represents that the data obtained from the group is normal.

Table S15 The normality tests of YLS signal intensity data in control group and three treatment groups of four cicada species

**Species**	Control group	Heat shock group	Helper group	Heat + Helpers group
Karenia caelatata	0.775	0.308	0.007	0.318
Hyalessa maculaticollis	0.931	0.940	0.047	0.686
Cryptotympana atrata	0.046	0.085	0.952	0.722
Macrosemia insignis	0.015	0.002	0.018	0.071

Note: The value more than 0.5 represents that the data obtained from the group is normal.

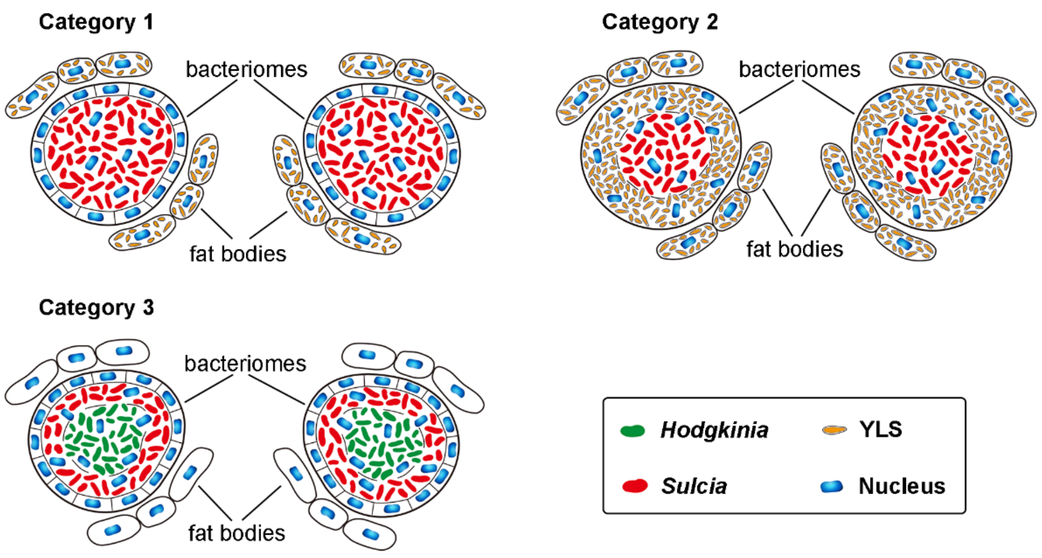

Categories	Representative cicada species
1.	***Karenia caelatata***, ***Cryptotympana atrata***, ***Macrosemia insignis***, *Subpsaltria yangi* *Huechys thoracica, Mogannia conica, Mogannia indigotea, Mogannia cyanea, Mogannia minuta* *Cryptotympana holsti, Cryptotympana facialis, Pomponia linearis, Dundubia hainanensis* *Macrosemia umbrata, Meimuna iwasakii, Meimuna kuroiwae, Meimuna mongolica* *Meimuna oshimensis, Meimuna opalifera, Purana gigas, Gaeana maculata* *Ambragaeana sticta, Tanna* sp., *Tanna japonensis*
2.	***Hyalessa maculaticollis***, *Graptopsaltria tienta*
3.	***Eopycna coelestia***, ***Eopycna repanda***, ***Platypleura kaempferi***, *Tettigades undata* *Tettigetta* sp., *Kosemia yezoensis, Muda kuroiwae, Katoa paucispina, Auritibicen jai* *Magicicada septendecim, Magicicada tredecim, Auritibicen japonicus, Auritibicen bihamatus*

Fig S1. Schematic representation showing the complex distribution of YLS, *Hodgkinia* and *Sulcia* in cicadas based on these seven representative cicada species and other cicada species.

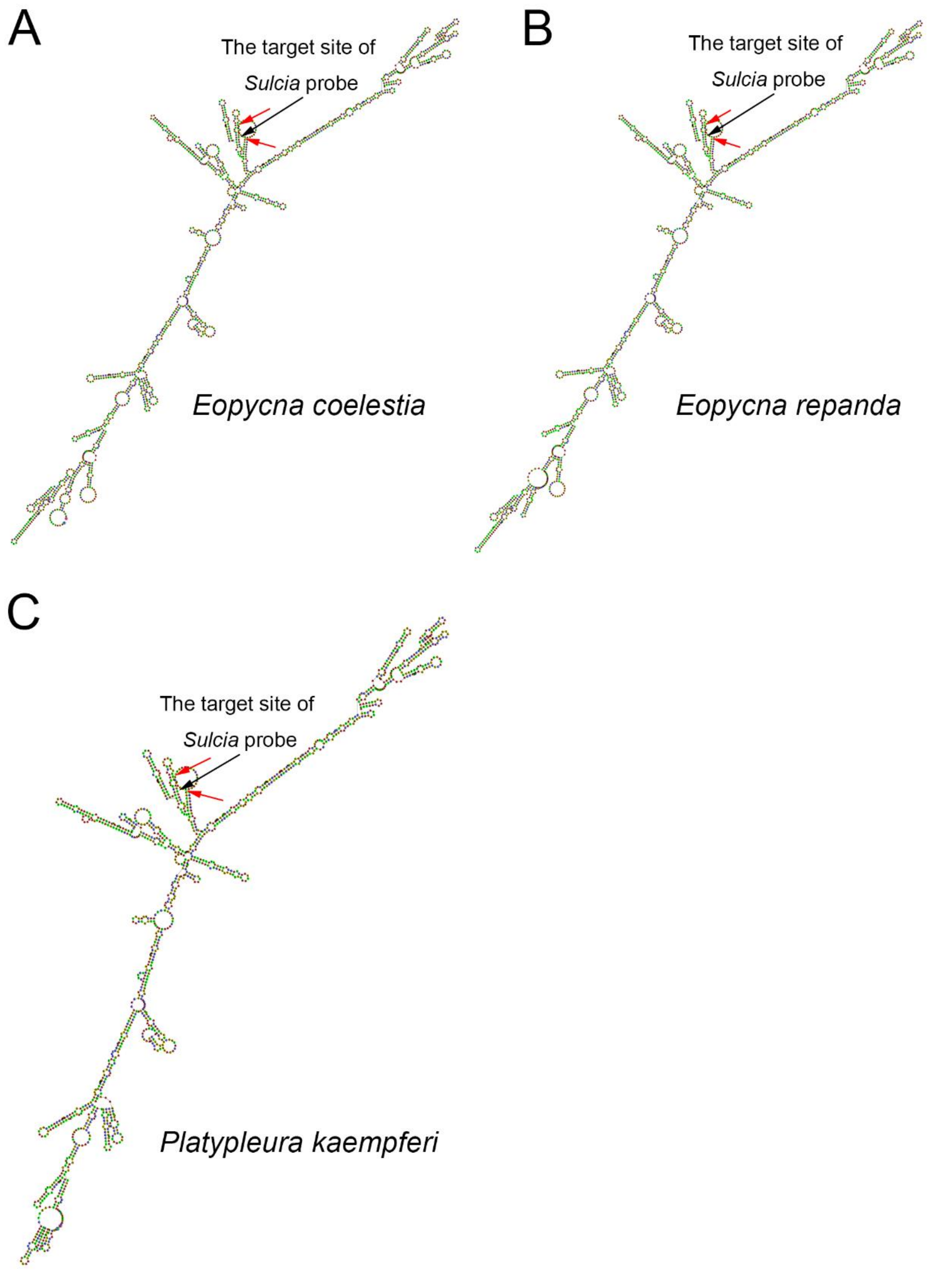

Fig S2. The predicted secondary structure of the 16S rRNA gene of *Sulcia* in *Eopycna coelestia*, *Eopycna repanda* and *Platypleura kaempferi*. Black arrows represent the target region of *Sulcia* probe. Red arrows represent the start/stop target site of *Sulcia* probe.

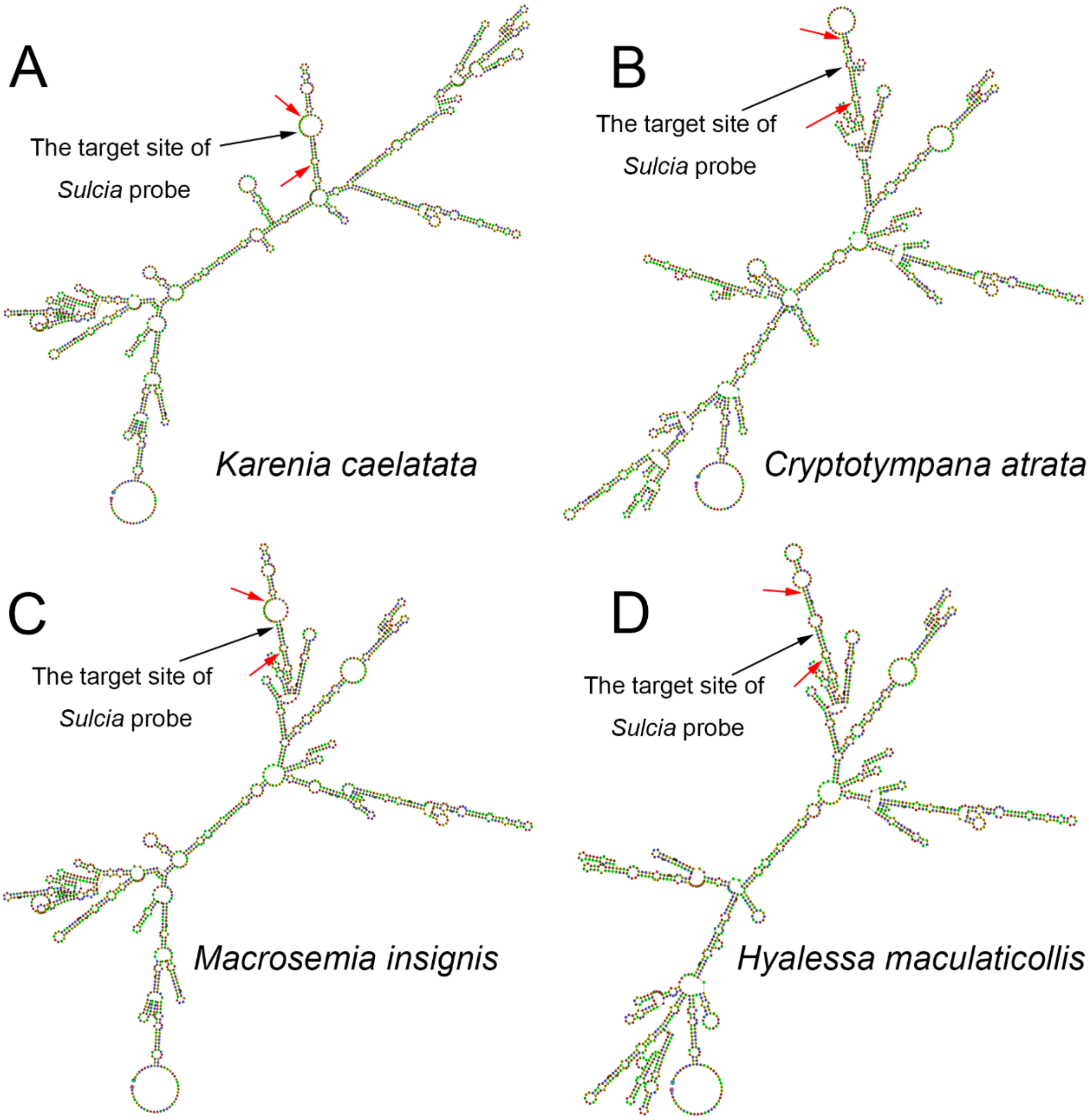

Fig S3. The predicted secondary structure of the 16S rRNA gene of *Sulcia* in *Karenia caelatata, Cryptotympana atrata, Macrosemia insignis and Hyalessa maculaticollis*. Black arrows represent the target region of *Sulcia* probe. Red arrows represent the start/stop target site of *Sulcia* probe.

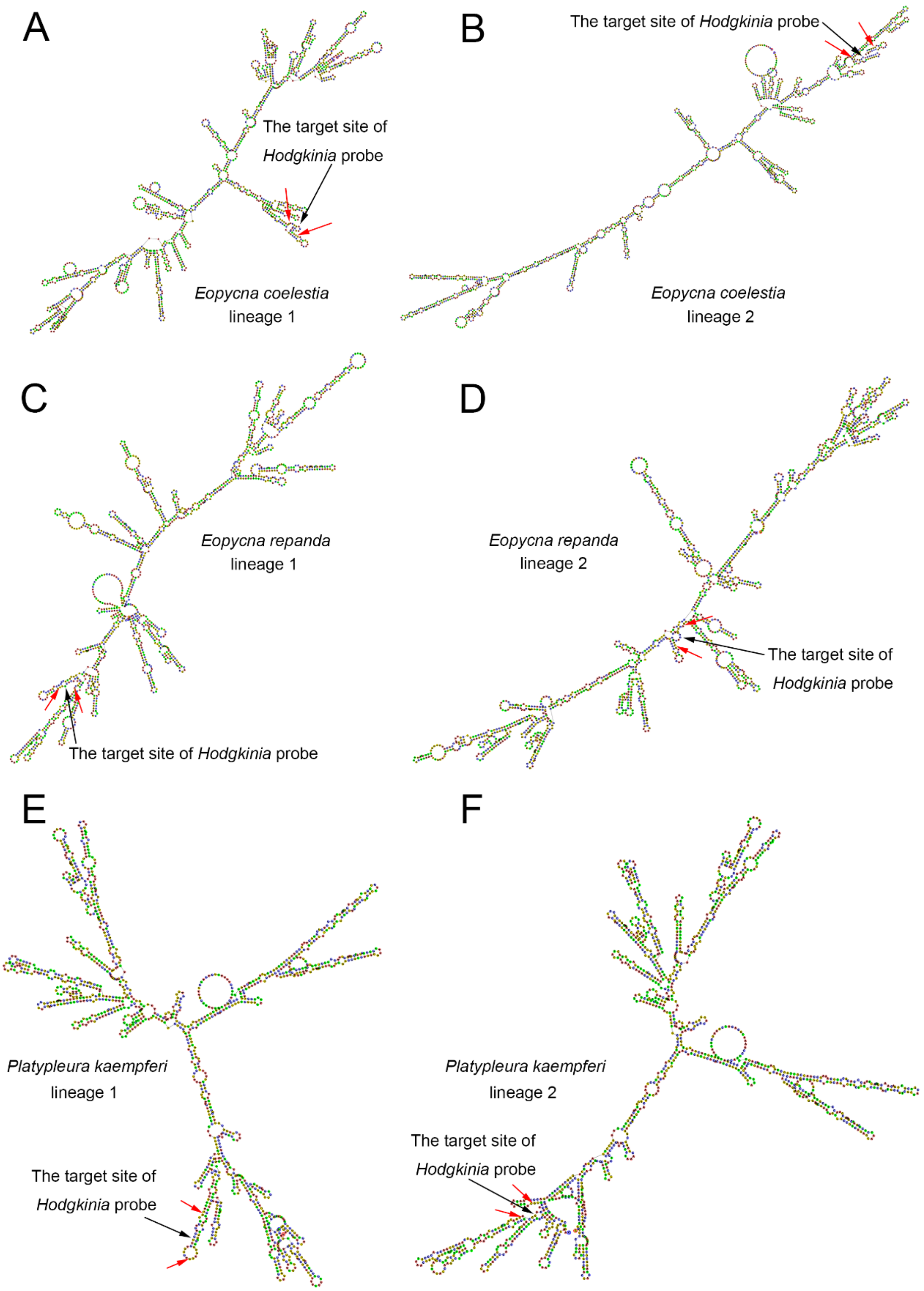

Fig S4. The predicted secondary structure of the 16S rRNA gene of representative *Hodgkinia* lineages in *Eopycna coelestia*, *Eopycna repanda* and *Platypleura kaempferi*. Black arrows represent the target region of *Hodgkinia* probe. Red arrows represent the start/stop target site of *Hodgkinia* probe.

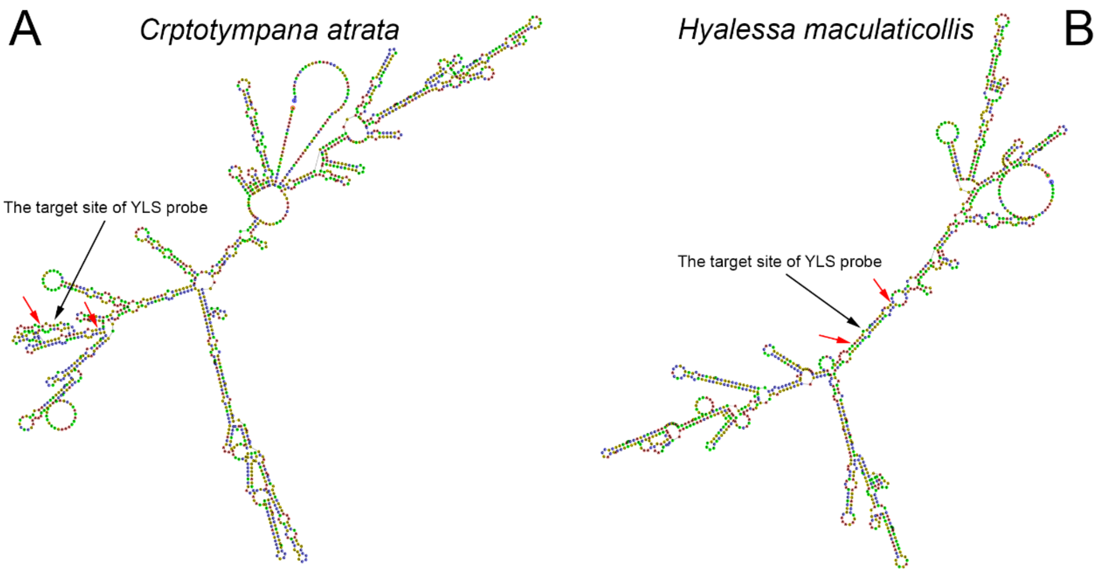

Fig S5. The predicted secondary structure of the 18S rRNA gene of YLS in *Cryptotympana atrata* and *Hyalessa maculaticollis*. Black arrows represent the target region of YLS probe. Red arrows represent the start/stop target site of YLS probe.

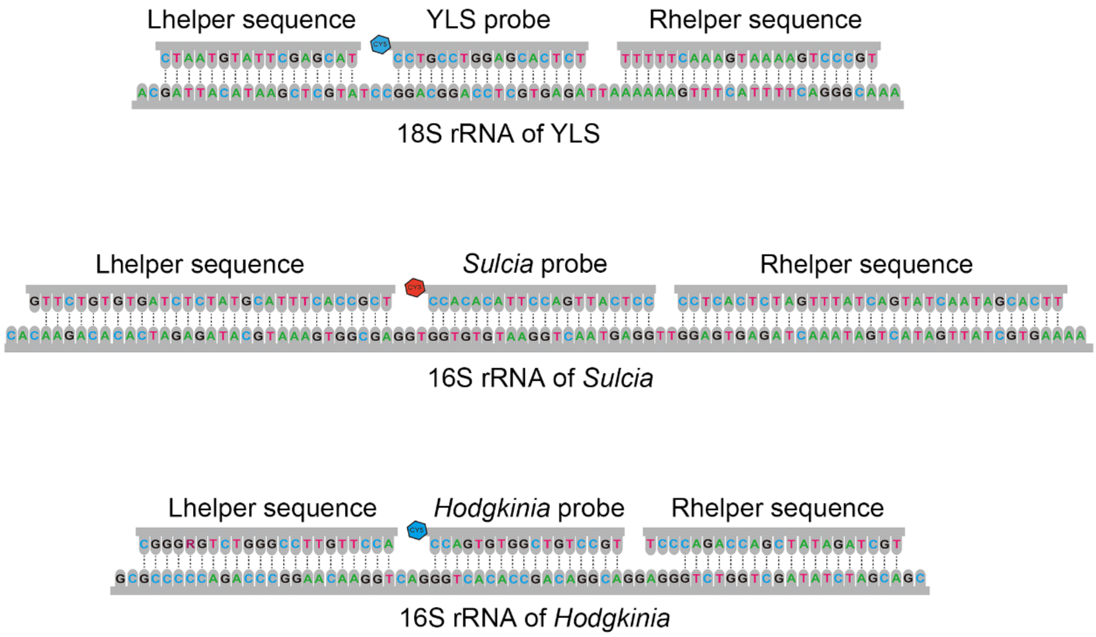

Fig S6. Schematic representation showing the probe binding sites and unlabeled oligonucleotides (helpers) binding sites of *Hodgkinia*, *Sulcia* and YLS in cicadas.
